# Michigan Dental Association

**Published:** 1876-01

**Authors:** W. D. Tremper, Thos. R. Perry


### MICHIGAN DENTAL ASSOCIATION.

The twentieth annual meeting of the Michigan Dental Association met at Ann Arbor, Oct. 12, and was called to order by the President Thos. R. Perry. On, motion the meeting adjourned to assemble in the Dental College Building at 2 p. m.

AFTERNOON SESSION.

Meeting called to order by the President at 2 p. m. First order of business payment of dues.

Moved by Dr. Spellman that Drs. Thos. Rex and J. A. Harris be recognized as members of this Association after payment of back dues. Carried.

The next in order was calling roll, which was followed by reports of Standing Committees. Dr. W. P. Morgan of Committee on Anatomy, read a paper on "muscles of the face."

Dr. D. C. Hawxhurst had a very interesting report on Hygiene.

Dr. Taft thinks too much importance relatively is attached to the merely physical conditions in this connection and mental influences too much overlooked. An excited condition of the mind of the mother will produce an influence on the milk often rendering it unfit for the child. He thinks the mother at such times should be kept as free from mental perturbations as possible.

Another erroneous idea is often entertained, viz: That the only thing to be attended to is supplying the mother with proper aliment. In many cases it is not digested and appropriated. The changes and modifications which food must necessarily pass, are numerous before it will go to build up the tissues of the child.

The mental and physical influences go together and deserve equal attention.

Dr. Holmes coincides with the other gentlemen. Thinks the mind is less liable to injurious influences when the body is in a healthy condition.

Adjourned to meet at 7 p. m.

Seven p. m. Meeting called to order by the President. Minutes of previous session read and approved. Dr. B. T. Spelman, Chairman of the committee to solicit contributions of dental manufactures for the Dental Department of Michigan University, reported progress. Report accepted and committee continued.

The subject of surgery was taken up.

Dr. Spelman, speaking of alveolar abscess, thinks it a disease of periosteum, generally arising from exposed pulps.

The nerve takes on pathological action, increased flow of blood, congestion, death and suppuration follow. Poisonous gases, resulting from decomposition of pulp, cause the same pathological action in surrounding parts. Exudation corpuseles which are thrown out to restore the tissues, are oxydized and decomposed and the result is an abscess. In treatment he closes nerve canal at once and treats through process.

Dr. Taft does not consider oxy-chloride of zinc the best material for root filling.

Dr. W. H. Jackson has met with little success in capping exposed nerves.

Dr. Spelman thinks they may be saved in a majority of cases. He leaves softened dentine over the nerve many times when it would otherwise be exposed.

Dr. Thos. Rex has dispensed almost entirely with the use of oxy chloride zinc. Uses cap of cork saturated with carvacrol and salicylic acid.

Dr. Douglass pumps creosote through the root till it is visible through the opening in the process. He fills root with gold.

He treats exposed nerves by removing all decayed dentine and applies a saturated solution of creosote and sulphate morphia; caps with a layer of gold foil, then oxy chloride of zinc.

Dr. Taft thinks the different susceptibilities of the pulps to injurious influences should be regarded.

Some pulps will die in spite of the most careful treatment after exposure, others are difficult to destroy.

He urges the preservation of the pulps whenever possible Thinks they should be restored as nearly as possible to the normal condition before capping.

For capping, use some material that will occupy all of the space, be about as good a conductor as dentine and be a non-irritating material. He thinks softened dentine may be in some cases just as good a capping as any other material.

Adjourned.

2D DAY, MORNING SESSION.

Wednesday, 9 a. m. Meeting came to order. Minutes of previous session read and with one correction, approved.

An invitation to the members of Michigan Dental Association to attend a special meeting of the New York Academy of Dental Surgery, was read and thanks extended for the same.

Dr Holmes offered a resolution of thanks to manufacturers of dental goods for valuable donations to the Dental Department of University of Michigan. Adopted.

Dr. G. R. Thomas presented a resolution to elect a visiting committee of three, to confer with the faculty of the new Dental Department upon such matters as might come before them. Adopted.

Committee, Drs. A. T. Metcalf, G. R. Thomas and J. W. Finch.

Dr. J. C. Parker moved that the Secretary be instructed to prepare a circular to be distributed among members of the profession soliciting contributions of interesting specimens for the purpose of founding a museum in connection with the Department of Dentistry of the University. Carried.

Adjourned.

House called to order at 2 p. m., by President Perry. President Angell, of the University, first addressed the meeting, expressing warm sympathy with the members of the Association in their efforts to establish a dental college.

He was followed by Prof. Ford, of the Medical Department of the University, who gave a very interesting lecture on the "structure of bone."

The discussion on surgery was resumed. Dr. Rex related a case of fracture of submaxillary. He restored parts to place by pressure and held them there by an appliance, which he exhibited, in two weeks, union was effected.

Drs. Perry and Parker described some experiments with regard to leakage of amalgam fillings.

Dr. Jackson read a paper on "dental education."

Dr. Taft followed with remarks on the same subject.

Moved that when this meeting adjourns, it adjourns to meet in Ann Arbor on the last week of March, 1876. Carried. Dr. Taft took this occasion to express gratitude for the confidence manifested toward him by this association in recommending him to the Regents for a position in the University. Adjourned.

Wednesday, 7 p. m. Meeting came to order, minutes read and approved,

Dr. B. T. Spelman moved that an appropriation of one hundred dollars be made from the Treasury of this Association, to the University of Michigan, for the use of the Department of Dentistry. Carried. The election of officers resulted as follows: For President, J. W. Finch; Vice-Presieent, A. T. Metcalf; Secretary, W. P. Morgan; Treasurer, E. S. Holmes; Censor, D. C. Hawxhurst. The Association then listened to the retiring President's address. Meeting adjourned.

W. D. TREMPER, Sec., THOS. R. PERRY, Pres.

